# Assessment of dietary nitrate supplementation: prevalence of use, knowledge, attitudes and beliefs among active Australians

**DOI:** 10.3389/fnut.2023.1291431

**Published:** 2023-11-02

**Authors:** Nicholas F. McMahon, Paige G. Brooker, Toby Pavey, Michael D. Leveritt

**Affiliations:** ^1^School of Human Movement and Nutrition Sciences, University of Queensland, St. Lucia, QLD, Australia; ^2^School of Exercise and Nutrition Sciences, Queensland University of Technology, Kelvin Grove, QLD, Australia

**Keywords:** dietary supplements, nitrate, knowledge, prevalence of use, exercise performance, survey

## Abstract

**Introduction:**

Use of nitrate as a dietary supplement has gained popularity among athletes and recreationally active individuals to enhance exercise performance. However, the prevalence and patterns of use, and knowledge of nitrate as a dietary supplement are unknown.

**Methods:**

Individuals (≥16y) completed a 42-item online questionnaire to collect (i) sociodemographic information; (ii) participation in activity and sport; (iii) nitrate supplementation use and reasons; (iv) attitudes and beliefs regarding information sources and the safety of nitrate as a dietary supplement; and (v) knowledge of dietary nitrate supplements.

**Results:**

In total, 1,404 active adults (66% female) took part in the study. Only about one in 10 respondents (11.9%) reported they had consumed dietary nitrate (“users”) in the past, most commonly as beetroot juice (31.3%). Over two-thirds (69.4%) of users could not correctly identify the correct timing of intake relative to performance time to best improve exercise performance, and most users (82.3%) were unsure of the contraindications to oral consumption of dietary nitrate supplements. Only 3.9% of users experienced adverse effects after ingesting dietary nitrate supplements. Among non-users, the most common reasons respondents selected for not using dietary nitrate supplements were “I do not think I need to” (70.2%) and “I have never thought about it” (69.2%).

**Discussion:**

There is evidence to support the efficacy of dietary nitrate intake in improving exercise performance. However, findings from this study suggest dietary nitrate is under-utilized. Educational messages that target dietary nitrate consumption should be targeted toward nutritionists, coaches, and exercise physiologists to bridge the gap between knowledge-to-practice.

## Introduction

According to the World Health Organization (WHO), almost 80% of the global population use dietary supplements (1), often motivated by improving health and immunity, losing weight, increasing muscle mass, or improving skin and hair (2). The use of supplements among active adults has also been well documented (3–9), and often used to enhance exercise performance, increase their tolerance for more intense training, or for better recovery and training adaptations (10, 11). Dietary supplements may be in the form of extracts or concentrates from plants or animal-derived ingredients (such as whey protein), available as capsules, tablets, liquids, soft gels, powders and bars (11).

Commercially available beetroot juice, often in the form of “shots,” are becoming more commonplace in Australia, and promoted for use to enhance exercise performance (12). These drinks provide an acute, concentrated dose of nitrate of (NO_3_^−^), purposefully designed as a more convenient and palatable option (13). We have previously reviewed the evidence regarding the efficacy of nitrate as an ergogenic aid (12). Findings from our systematic review and meta-analysis of 76 trials across 47 studies suggest that dietary nitrate supplementation is likely to elicit a positive outcome when testing endurance exercise capacity but is less likely to be effective for time-trial performance. Doses of nitrate varied between studies from 4.1 mmol (~254 mg) to 19.5 mmol (~1,209 mg) administered on each trial day, and beetroot juice was the most common supplement source. Findings from our review have also been substantiated by more recent reviews, such as that of Senefeld et al. (14). While it is unlikely that consumption of beetroot juice or other vegetable sources of nitrate is harmful (and may, in fact, offer other health benefits), chronic use of nitrate supplements has not been well studied (12).

Anecdotally, the use of nitrate-rich beetroot juice was used extensively, and with significant benefit, by elite athletes from prominent countries competing at the 2012 London Olympic and Paralympic Games (15). However, it is still unclear if the use of dietary nitrate as a form of supplementation is widespread and how the knowledge from research studies is being translated. Despite the recent body of evidence supporting the ergogenic effect of dietary NO_3_^−^ on exercise performance, little is known about the knowledge and use of NO_3_^−^ as a supplement among active adults.

There is widespread availability of supplements, but studies evaluating the prevalence of their use, knowledge, attitudes and beliefs among consumers are scarce (2). Dietary supplements are available for purchase from supermarkets, pharmacies and fitness centers, without any medical prescription (16), and can lead to misuse. Social media has become a popular medium to share nutrition information, and individuals have high levels of trust in the information they see online (17). However, health misinformation on social media is widespread, particularly in relation to nutrition (18). Advertising products on social media, particularly supplements, is ubiquitous (19). Dietary NO_3_^−^ supplements are relatively new on the market, little is known about the “knowledge-to-action” gap (20). Therefore, the objectives of this study were to: (i) assess the prevalence of use of dietary NO_3_^−^ supplements among a sample of active adults; (ii) understand the frequency and pattern of their use; (iii) assess self-reported beliefs on the efficacy of dietary NO_3_^−^ supplementation as an ergogenic aid; (iv) investigate consumers motivations for using or abstaining from dietary NO_3_^−^ supplementation; and (v) explore the differences in NO_3_^−^ use between different socio-demographic characteristics.

## Materials and methods

### Participants and recruitment

This study was approved by the Human Research Ethics Committee of The University of Queensland – Approval number: HMS16/1210. Participants were recruited through print and media advertising via word of mouth and snowball sampling. Posters and flyers were distributed via university notice boards, mailing lists, cycling clubs/cafes, fitness and health centers, cafes, and a study website. Interested participants were encouraged to contact the lead investigator and were provided with more information about the study and had their questions answered. Individuals were considered eligible if they were healthy and active (≥150 min per week of moderate to vigorous physical activity by self-report) adults aged ≥16 years. Participants were excluded from the study if they (1) had history of clinical illness or disease such as new or possible symptoms; (2) were currently being treated with a diuretic; (3) had smoked cigarettes or quit smoking in the last 3 months; (4) had a body mass index below 18.5 kg/m^2^ or above 34.9 kg/m^2^; (5) were insufficiently active (<150 min per week of moderate to vigorous physical activity); (6) were unable to read or speak English; (7) were pregnant or lactating.

Completion and submission of the questionnaire was considered as informed consent and responses were anonymous. After completing the questionnaire, participants had the option to provide their email address to be entered into a draw to win one of four gift cards. Where participants opted into the draw, questionnaire responses were not attributable to the participant’s identifying information.

### Questionnaire design

A multicomponent questionnaire was designed to investigate dietary NO_3_^−^ usage, practices, attitudes and beliefs in a sample of active Australians. The questionnaire was built using an online survey tool (SurveyMonkey Inc., California, United States) and only available in English. Participants were asked to choose the best response from the options provided. Where applicable, participants could also use the text field to provide open responses or to elaborate on information they provided.

A copy of the preliminary questionnaire was given to 10 accredited allied health professionals for feedback on the questionnaires’ content, length and language, and feedback was integrated into the design process. The questionnaire took approximately 3–10 min to complete, which was considered suitable by the health professionals.

The final confirmed questionnaire included questions regarding dietary NO_3_^−^ supplementation and included whether participants are currently consuming dietary NO_3_^−^ supplements and, if so, in what form and type. Further quantitative and descriptive questions included: (1) demographic data (age, ethnicity, sex); (2) definition of supplement, knowledge on legality based on the participants’ understanding, and NO_3_^−^ rich sources; (3) consumption frequency and history of use; (4) rationale for NO_3_^−^ supplementation (attitudes and beliefs); (5) experience with the supplement (positive or negative and any side effects); (6) sources of NO_3_^−^ supplement advice/information; (7) training type and volume (hours/week); (8) performance level; (9) motivation and aspirations; and (10) any negative effects experienced after dietary NO_3_^−^ supplementation. The final part of the questionnaire included a dietary NO_3_^−^ supplementation knowledge quiz. It comprised six multiple-choice questions which were concerned with the general knowledge of dietary NO_3_^−^ supplementation (Supplementary material).

Before being disseminated to study participants, the final confirmed questionnaire was pilot-tested by a sample of 20 active adults using a test and retest format. The sample group was given the questionnaire on two different occasions (7-days apart). The level of agreement between reviewers evaluating the reliability of the questionnaire was assessed using Cohen’s kappa statistics. The kappa values were interpreted using the ranges suggested by Landis and Koch (21) of <0.00 = poor, 0.00–0.20 = slight, 0.21–0.40 = fair, 0.41–0.60 = moderate, 0.61–0.80 = substantial, 0.81–1.00 = almost perfect. The kappa score was 0.932, suggesting the questionnaire had substantial to almost perfect interrater reliability.

### Statistical analysis

At the time of the study, there were approximately 2,762,000 sufficiently active adults in Australia (22). Using the sample size calculator (Creative Research Systems, Petaluma, CA), it was calculated that 384 participants were needed to represent the population. Based on an anticipated response rate of 60%, it was calculated that 640 participants were needed to maintain statistical significance. Associations between each variable were coded and assessed by chi-square (χ^2^) analysis using SPSS for Windows (Version 23.0, Armonk, NY: IBM Corp.). Participants that were identified as “unsure” users were excluded from chi-square (χ^2^) comparisons. An independent samples *t*-test was conducted to determine if dietary NO_3_^−^ supplement use influenced other variables. Statistical significance was set at *p* ≤ 0.05. All data are expressed as mean ± standard deviation unless otherwise stated.

## Results

One-thousand four-hundred and four (1,404) individuals took part in the study (66% female); of which, 1,177 (84%) completed the questionnaire. Just over half (57.7%) of the participants were between the age of 16–25 years. Most participants considered themselves Australian (73.7%), having a healthy body mass index (≥18.5–24.99 kg/m^2^; 66.7%) and had completed secondary school or above (69.8%). The percentage of those taking part in over 2.5 h of exercise was 65.9, and 68.8% reported exercising ≥3 times per week. The most popular forms of exercise were running (24.4%) and going to the gym (22.3%). Most participants’ current sporting level was “recreational” (71.7%) and 5.2% (*n* = 69) were performing at either a national or international level. The demographic characteristics of participants are presented in Table 1.

**Table 1:** Demographic characteristics.

	Users	%	Non-users	%	Total	%
**Sex**	*x*^2^ = 9.139; *p* = 0.003					
Men	71	5.2%	363	26.4%	459	33.4%
Women	92	6.7%	784	57.1%	914	66.6%
**Ages**	*x*^2^ = 17.877; *p* = 0.057					
16–20	49	3.7%	428	32.7%	477	36.4%
21–25	39	3.0%	253	19.3%	292	22.3%
26–30	19	1.5%	96	7.3%	115	8.8%
31–35	20	1.5%	68	5.2%	88	6.7%
36–40	11	0.8%	95	7.3%	106	8.1%
41–45	11	0.8%	62	4.7%	73	5.6%
46–50	8	0.6%	64	4.9%	72	5.5%
51–55	5	0.4%	40	3.1%	45	3.4%
56–60	0	0.0%	19	1.5%	19	1.5%
61–65	1	0.1%	16	1.2%	17	1.3%
66+	0	0.0%	6	0.5%	6	0.5%
**Ethnicity**	*x*^2^ = 9.914; *p* = 0.271					
Australian	115	8.8%	849	64.8%	964	73.6%
Aboriginal or Torres Strait Islander	1	0.1%	8	0.6%	9	0.7%
United Kingdom	4	0.3%	49	3.7%	53	4.0%
Italian	1	0.1%	8	0.6%	9	0.7%
German	0	0.0%	9	0.7%	9	0.7%
Chinese	15	1.1%	79	6.0%	94	7.2%
Indian	2	0.2%	21	1.6%	23	1.8%
Greek	0	0.0%	6	0.5%	6	0.5%
Other	25	1.9%	118	9.0%	143	10.9%
**BMI** (**kg·m**^**−2**^)	*x*^2^ = 1.673; *p* = 0.796					
<18.5	12	0.9%	82	6.3%	94	7.2%
≥18.5–24.99	104	8.0%	762	58.4%	866	66.4%
≥25–29.99	34	2.6%	237	18.2%	271	20.8%
≥30–39.99	11	0.8%	52	4.0%	63	4.8%
>40	1	0.1%	9	0.7%	10	0.8%
**Education**	*x*^2^ = 10.952; *p* = 0.141					
Some secondary school	2	0.2%	20	1.5%	22	1.7%
Year 12	52	4.0%	456	34.8%	508	38.8%
Trade or apprenticeship	4	0.3%	20	1.5%	24	1.8%
TAFE	20	1.5%	131	10.0%	151	11.5%
Bachelor degree	51	3.9%	358	27.3%	409	31.2%
Postgraduate diploma	8	0.6%	51	3.9%	59	4.5%
Master’s degree	20	1.5%	70	5.3%	90	6.9%
Doctorate	6	0.5%	41	3.1%	47	3.6%
**Income**	*x*^2^ = 14.031; *p* = 0.172					
$0–$9,999	44	3.4%	365	27.9%	409	31.2%
$10,000–$19,999	28	2.1%	179	13.7%	207	15.8%
$20,000–$29,999	9	0.7%	123	9.4%	132	10.1%
$30,000–$39,999	9	0.7%	51	3.9%	60	4.6%
$40,000–$49,999	8	0.6%	34	2.6%	42	3.2%
$50,000–$59,999	11	0.8%	41	3.1%	52	4.0%
$60,000–$79,999	12	0.9%	78	6.0%	90	6.9%
$80,000–$99,999	14	1.1%	74	5.6%	88	6.7%
$100,000–$149,999	13	1.0%	65	5.0%	78	6.0%
$150,000+	4	0.3%	35	2.7%	39	3.0%
Prefer not disclose	11	0.8%	102	7.8%	113	8.6%
**Are you vegetarian**	*x*^2^ = 4.994; *p* = 0.417					
No	143	10.9%	1,043	79.6%	1,186	90.5%
Yes-Lacto-ovo	7	0.5%	43	3.3%	50	3.8%
Yes-Lacto-veg	2	0.2%	4	0.3%	6	0.5%
Yes-Ovo-veg	2	0.2%	5	0.4%	7	0.5%
Yes-Vegan	4	0.3%	34	2.6%	38	2.9%
Other	5	0.4%	18	1.4%	23	1.8%
**Amount of exercise**	*x*^2^ = 1.745; *p* = 0.187					
>2.5 h	99	7.6%	757	57.8%	856	65.3%
<2.5 h	64	4.9%	390	29.8%	454	34.7%
**Exercise type**	*x*^2^ = 26.493; *p* = 0.047					
Athletics	5	0.4%	30	2.3%	35	2.7%
Swimming	6	0.5%	23	1.8%	29	2.2%
Basketball	1	0.1%	24	1.8%	25	1.9%
Cycling	8	0.6%	34	2.6%	42	3.2%
Running	42	3.2%	274	20.9%	316	24.1%
Netball	2	0.2%	51	3.9%	53	4.0%
Rugby	3	0.2%	24	1.8%	27	2.1%
AFL	3	0.2%	14	1.1%	17	1.3%
Gym-goer	48	3.7%	246	18.8%	294	22.4%
Soccer	4	0.3%	41	3.1%	45	3.4%
Rowing	4	0.3%	9	0.7%	13	1.0%
Marathon running	4	0.3%	20	1.5%	24	1.8%
Triathlon	2	0.2%	18	1.4%	20	1.5%
Surf life-saving	0	0.0%	3	0.2%	3	0.2%
Walking	9	0.7%	122	9.3%	131	10.0%
I do not do any exercise	5	0.4%	36	2.7%	41	3.1%
Other	17	1.3%	178	13.6%	195	14.9%
**Exercise frequency**	*x*^2^ = 12.485; *p* = 0.014					
Sporadic	9	0.7%	109	8.6%	118	9.3%
1–2 times	22	1.7%	257	20.3%	279	22.0%
3–4 times	76	6.0%	464	36.6%	540	42.6%
5–6 times	36	2.8%	213	16.8%	249	19.6%
Daily	15	1.2%	68	5.4%	83	6.5%
**Years of participation**	*x*^2^ = 7.158; *p* = 0.128					
<1	8	0.6%	95	7.5%	103	8.1%
1–2 years	42	3.3%	215	16.9%	257	20.3%
3–4 years	36	2.8%	225	17.7%	261	20.6%
5–6 years	16	1.3%	124	9.8%	140	11.0%
7+ years	56	4.4%	452	35.6%	508	40.0%
**Current sporting level**	*x*^2^ = 3.033; *p* = 0.552					
Recreational	111	8.7%	796	62.7%	907	71.5%
Club level	28	2.2%	206	16.2%	234	18.4%
State	7	0.6%	53	4.2%	60	4.7%
National	10	0.8%	39	3.1%	49	3.9%
International	2	0.2%	17	1.3%	19	1.5%
**How important highest possible standard**	*x*^2^ = 17.049; *p* = 0.004					
Very important	28	2.2%	119	9.4%	147	11.6%
Important	40	3.2%	219	17.3%	259	20.4%
Mod. Important	28	2.2%	179	14.1%	207	16.3%
Slightly important	16	1.3%	93	7.3%	109	8.6%
Not important	11	0.9%	135	10.6%	146	11.5%
Own expectations	35	2.8%	366	28.8%	401	31.6%

BMI, body mass index.

### Prevalence and patterns of use of nitrate as a dietary supplement

About one in ten (11.9%) participants reported they had consumed dietary nitrate (“users”) in the past, most commonly in the form of beetroot juice (31.3%; Table 2). When asked about typical servings, most users (66.2%) reported having “one to three” servings per week, with most opting for an acute dosage immediately before the event/sport/exercise (42.1%). There was a significant relationship found between gender and use of dietary NO_3_^−^ supplements, with greater usage in males (15.5% vs. 10.1% for females; *x*^2^ = 9.139, *p* = 0.003).

**Table 2:** Nitrate supplement usage.

	Users	%	Unsure	%	Total	%
**In total, how long have you been using dietary nitrate supplements for?**						
<month	43	27.9%	15	9.7%	58	37.7%
2–6 months	36	23.4%	4	2.6%	40	26.0%
7 months–1 year	15	9.7%	1	0.6%	16	10.4%
1–2 years	20	13.0%	2	1.3%	22	14.3%
>2 years	15	9.7%	3	1.9%	18	11.7%
**Type of dietary nitrate supplement used**						
Beetroot juice	53	29.1%	4	2.2%	57	31.3%
BeetIT sports bar	22	12.1%	3	1.6%	25	13.7%
Powder	38	20.9%	4	2.2%	42	23.1%
Capsule	20	11.0%	4	2.2%	24	13.2%
Whole beetroot	2	1.1%	0	0.0%	2	1.1%
Other	28	15.4%	4	2.2%	32	17.6%
**Amount/dose typically consumed**						
1 × 70 mL BEET shot	30	19.5%	5	3.2%	35	22.7%
1 × 40 g BEET bar	12	7.8%	4	2.6%	16	10.4%
1 × 250 mL beetroot juice	18	11.7%	1	0.6%	19	12.3%
1 × Tbsp beet powder	22	14.3%	2	1.3%	24	15.6%
2 × iForce capsules	13	8.4%	2	1.3%	15	9.7%
Other	34	22.1%	11	7.1%	45	29.2%
**Servings per week (1 serving = amount indicated on the product label)**						
1–3 times	85	55.2%	17	11.0%	102	66.2%
4–6 times	35	22.7%	4	2.6%	39	25.3%
7–9 times	5	3.2%	2	1.3%	7	4.5%
10–12 times	2	1.3%	1	0.6%	3	1.9%
13+ times	2	1.3%	1	0.6%	3	1.9%
**If used for exercise performance when do/did you take dietary nitrate supplements?**						
Several days before event	34	17.4%	6	3.1%	40	20.5%
Immediately before event	72	36.9%	10	5.1%	82	42.1%
During event	11	5.6%	4	2.1%	15	7.7%
Immediately after event	19	9.7%	7	3.6%	26	13.3%
In the hours after the event	25	12.8%	3	1.5%	28	14.4%
Other	3	1.5%	1	0.5%	4	2.1%
**Where did you obtain/buy your dietary nitrate supplements?**						
Team sponsor	3	1.6%	1	0.5%	4	2.2%
Nutritionist/dietitian	11	6.0%	0	0.0%	11	6.0%
Pharmacy	34	18.5%	6	3.3%	40	21.7%
Teammate	3	1.6%	0	0.0%	3	1.6%
Family member	9	4.9%	2	1.1%	11	6.0%
Coach	7	3.8%	1	0.5%	8	4.3%
Internet	35	19.0%	6	3.3%	41	22.3%
Retail	46	25.0%	8	4.3%	54	29.3%
Other	8	4.3%	4	2.2%	12	6.5%
**Do you think there are any risks (health or otherwise) associated with dietary nitrate supplements?**						
Yes	30	19.5%	7	4.5%	37	24.0%
No	58	37.7%	2	1.3%	60	39.0%
Unsure	41	26.6%	16	10.4%	57	37.0%
**Have you ever experienced adverse effects from taking dietary nitrate supplements?**						
Yes	5	3.2%	2	1.3%	7	4.5%
No	124	80.5%	23	14.9%	147	95.5%
**What were the symptoms?**						
Abdominal pain	0	0.0%	0	0.0%	0	0.0%
Beeturia (red or pink urine)	2	13.3%	1	6.7%	3	20.0%
Red stools	3	20.0%	1	6.7%	4	26.7%
Nausea and/or vomiting	0	0.0%	1	6.7%	1	6.7%
Headache	0	0.0%	1	6.7%	1	6.7%
Gastrointestinal distress	5	33.3%	0	0.0%	5	33.3%
Chest pain	0	0.0%	0	0.0%	0	0.0%
Disturbed sleep	0	0.0%	0	0.0%	0	0.0%
Other	0	0.0%	1	6.7%	1	6.7%
**Did you continue to take the supplement despite the adverse effects?**						
Yes	4	57.1%	1	14.3%	5	71.4%
No	1	14.3%	1	14.3%	2	28.6%

### Reasons for taking dietary nitrate supplements

**Figure 1:**
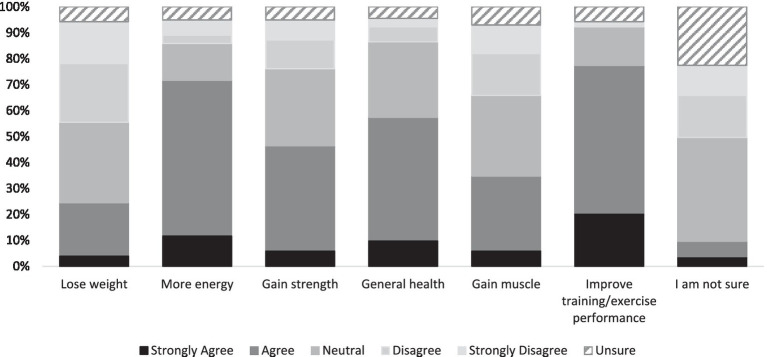
Reasons for taking dietary nitrate supplements. Data presented are the participants’ level of agreement with the reasons presented, expressed as a percentage.

**Figure 2:**
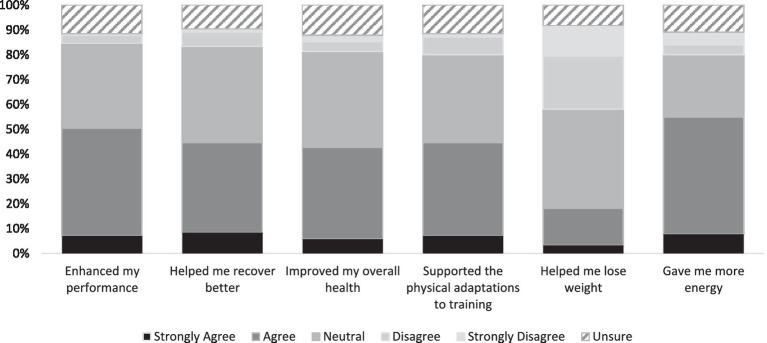
Experience using dietary nitrate supplements. Data presented are the participants’ level of agreement with the statements presented, expressed as a percentage.

Overall, participants reported “more energy” (71.6%) and “to improve training/exercise performance” (77.4%) as their rationale for taking dietary NO_3_^−^ supplements (Figure 1). Over half of users reported having “more energy” (54.8%) and “enhanced performance” (50.3%) after ingesting the supplement (Figure 2).

### Perceived safety of use of nitrate as a dietary supplement

About four in ten (39%) participants believe there are no health risks in supplementing their diets with NO_3_^−^, however, 37% admitted to being unsure about the safety of the supplement, but still took them anyway. Seven of the 154 users (4.5%) had experienced adverse effects from taking dietary NO_3_^−^ supplements with symptoms including beeturia (red or pink urine) (*n* = 3), red stools (*n* = 4), gastrointestinal distress (*n* = 5), and 1 each for nausea and/or vomiting, headache, and lightheaded. Participants could select more than one symptom (Table 2).

### Reasons for not using dietary nitrate supplements

**Figure 3:**
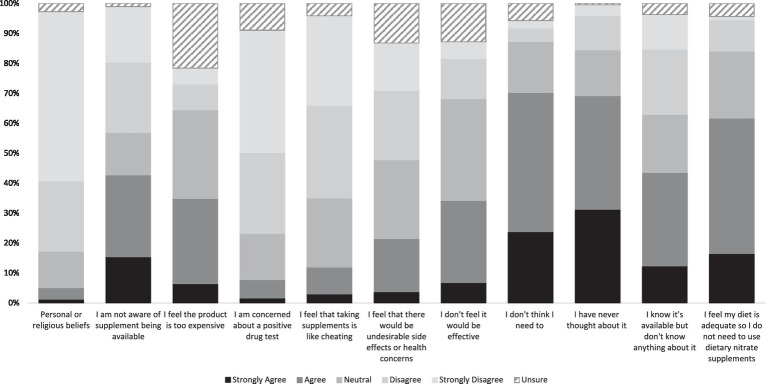
Reasons for non-use of dietary nitrate supplements. Data presented are the participants’ level of agreement with the reasons presented, expressed as a percentage.

Of the 1,115 participants reporting they had not used NO_3_^−^ supplements (“non-users”) in the past, 42.3% reported they were unaware of its availability. Over one third of non-users reported they did not feel like dietary nitrate supplements were effective, and 60% felt they did not need dietary NO_3_^−^ supplements because their diet was adequate (Figure 3).

Participants also reported that they were unsure if supplementing their diets with nitrate caused undesirable side effects or health conditions (25.3%). However, the majority (65.9%) reported they were not concerned about a positive drug test after supplementing with dietary NO_3_^−^.

### Influence on supplement use

**Figure 4:**
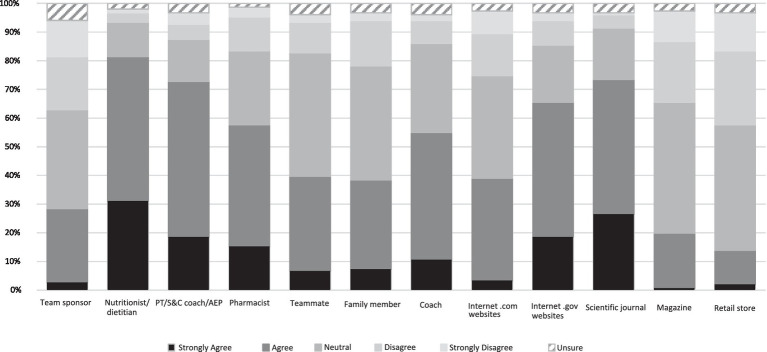
Influence of different information sources on participants’ supplement use. Data presented are the participants’ level of agreement with the information sources presented, expressed as a percentage. Abbreviations: PT, personal trainer; S&C, sports and conditioning; AEP, accredited exercise physiologist.

**Figure 5:**
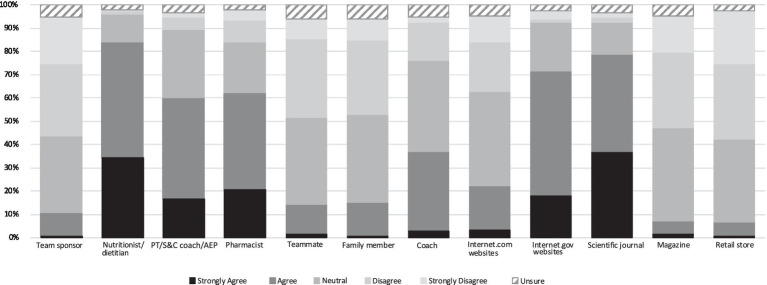
Perceptions of the credibility of different information sources. Data presented are the participants’ level of agreement with the information sources presented, expressed as a percentage. Abbreviations: PT, personal trainer; S&C, sports and conditioning; AEP, accredited exercise physiologist.

Participants identified various sources when asked what would influence their supplement use. Nutritionists and dietitians had the greatest influence (81.5%) followed by scientific journals (73.5%) and personal trainer/strength and conditioning coach/exercise physiologist (72.9%). Magazines (19.9%) and retail stores (13.9%) were reported as having the least amount of influence (Figure 4). Similar results were found when participants were asked what would provide the most credible source of information regarding supplement use with nutritionists and dietitians proving to be the most credible (84.1%), followed by scientific journals (78.8%) and government websites (71.5%). Retail stores (6.6%) and magazines (7.3%) were reported as the less credible sources of important regarding supplement usage (Figure 5).

### Dietary nitrate knowledge

Table 3 shows the participants’ self-reported level of knowledge regarding dietary NO_3_^−^ supplementation. Approximately 20% of participants correctly identified (or guessed) the Australian Institute ABCD classification for nitrate (group A). Only 6.3% of participants could correctly identify that antibacterial mouthwash reduces the effectiveness of dietary NO_3_^−^ supplements. Regarding the most appropriate use for nitrate supplementation, 14.3% of participants correctly identified that beetroot juice has been shown to be most effective 2–3 h prior to exercise.

**Table 3:** Dietary nitrate knowledge.

	Users	%	Non-users	%	Total	%
**Beetroot juice/nitrate is a group ____ Supplement**						
	*x*^2^ = 17.316; *p* = 0.002					
A (correct answer)	41	3.5%	212	18.0%	253	21.5%
B	49	4.2%	386	32.7%	435	36.9%
C	7	0.6%	117	9.9%	124	10.5%
D	1	0.1%	3	0.3%	4	0.3%
Do not know	26	2.2%	337	28.6%	363	30.8%
**Prior to this survey did you know beetroot juice had a particularly high nitrate content?**						
	x^2^ = 40.068; *p* = 0.000					
Yes (correct answer)	75	6.4%	336	28.5%	411	34.9%
No	49	4.2%	719	61.0%	768	65.1%
**What can reduce the effectiveness of oral consumption of dietary nitrate supplements?**						
	*x*^2^ = 46.055; *p* = 0.000					
Caffeine	31	2.6%	188	15.9%	219	18.6%
Mouthwash (correct answer)	22	1.9%	52	4.4%	74	6.3%
Fruit	8	0.7%	28	2.4%	36	3.1%
Do not know	63	5.3%	787	66.8%	850	72.1%
**The level of nitrate in a Beet-It sport shot can be achieved by consuming a small amount of nitrate-rich vegetables (spinach, rocket, lettuce, and beetroot)?**						
	*x*^2^ = 12.638; *p* = 0.002					
Yes (correct answer)	36	3.1%	252	21.4%	288	24.4%
No	29	2.5%	143	12.1%	172	14.6%
Do not know	59	5.0%	660	56.0%	719	61.0%
**According to scientific research, commercially available beetroot juice has been shown to be most effective ___ hours prior to exercise for immediate results?**						
	*x*^2^ = 58.169; *p* = 0.000					
2–3 h (correct answer)	38	3.2%	131	11.1%	169	14.3%
0.5–1 h	25	2.1%	141	12.0%	166	14.1%
24 h	9	0.8%	19	1.6%	28	2.4%
Do not know	52	4.4%	764	64.8%	816	69.2%
**Dietary nitrate supplements are most likely to improve your**						
	*x*^2^ = 44.895; *p* = 0.000					
100 m sprint	4	0.3%	35	3.0%	39	3.3%
1-rep max	2	0.2%	14	1.2%	16	1.4%
TTE (correct answer)	42	3.6%	160	13.6%	202	17.1%
All of the above	37	3.1%	206	17.5%	243	20.6%
Do not know	39	3.3%	640	54.3%	679	57.6%

## Discussion

In the past decade, there has been a plethora of research (>100 trials) investigating the role of dietary nitrate. Despite this, to date, there had been no published papers which have collected data on real-world usage. The primary purpose of the study was to determine the use of dietary NO_3_^−^ usage, the reason/s for use, and to compare knowledge and beliefs among active adults. The findings of this study showed the relative under-use of nitrate as a dietary supplement, with only 12% of the total sample reporting they had tried it, and of these, 61.2% had used the supplement for a period shorter than 6 months. There was a significant relationship found between gender and use of dietary NO_3_^−^ supplements, with greater usage in males. This finding is congruent with the literature which shows males place more emphasis on performance. However, general supplement use is higher in women, largely driven by their interest in improving overall health (23–29), whereas males place more of an emphasis on athletic performance-enhancing factors (5, 9, 30). There has been a significant focus in recent years on dietary NO_3_^−^ research tailored toward performance outcomes, however with research shifting toward health outcomes, positive results could create a shift in the gender gap.

Until recently, NO_3_^−^ was grouped with the other N-nitroso compounds and was associated with several negative health outcomes (31–36) which may also partly explain the relatively low prevalence of use supported by our data, whereby 24% of those surveyed thought there were risks associated with taking dietary NO_3_^−^ supplements. Of the dietary NO_3_^−^ supplement users in the present study, 3.9% reported they had experienced minor adverse effects. Associated side-effects included beeturia (*n* = 2), red stools (*n* = 3), and gastrointestinal distress (*n* = 5). Similar effects were reported by McMahon et al. (12) and Stanaway et al. (37). No chronic or major adverse events were reported across the 59 studies or in the current population.

The most common information source for participants came from nutritionists, dietitians, exercise professionals and/or scientific journals. However, the knowledge section of the survey showed a limited understanding. While general knowledge of current dietary NO_3_^−^ recommendations is not essential for every user (especially high-level athletes that place their trust in professionals working with them), it is important to be informed so that the best potential outcome can be achieved. Only 17.7% of users knew antibacterial mouthwash nullifies the effect of dietary NO_3_^−^ supplementation. This is problematic because antibacterial mouthwash stops NO_3_^−^ reductase activity of oral bacteria, resulting in the elimination of NO_2_^−^ formation in the saliva (38). However, this lack of understanding comes back to the information source, and Shannon et al. (39) found that nutrition professionals’ knowledge about dietary NO_3_^−^ was generally poor. It is hard to counterbalance incorrect information or misconceptions if those deemed the most reliable are unsure themselves. The confusion and lack of knowledge may be because of a nutritional supplement space that has grown rapidly, so an emphasis needs to be placed on adapting to information rapidly, efficiently, and effectively to achieve the best outcomes.

Educating support staff (e.g., nutritionists, coaches, exercise physiologists) is a useful strategy to improve the dissemination of nitrate knowledge, but this may be limited to individuals with access to such a support network, i.e., athletes. Harnessing the power and influence of social media could be a useful health-promotion tool to reach broader audiences (19). Behavior change is complex, and improving knowledge alone may not be sufficient in altering individuals’ use of nitrate supplements. Drawing from the behavior change wheel framework to design and evaluate interventions aimed at increasing the use of nitrate supplementation could be useful. At the core of the behavior change wheel is a model for behavior knowns as the COM-B model: Capability, Opportunity, and Motivation-Behavior (40). The COM-B model could be used to explore perceived barriers and facilitators to identify potential levers for change for adoption of nitrate supplementation to occur (41).

The strength of our research is the examination and new information on dietary NO_3_^−^ usage, beliefs and knowledge. This could lead to the ability to access knowledge translation not only in dietary NO_3_^−^ but also to supplement use and research in general. By providing new information, future research can focus on clear guidelines and a way to translate knowledge effectively. However, the study has several notable limitations. Most participants were young (≤25 years of age; 58.7%), low income (<$30,000 annual income; 57.1%) and recent secondary school graduates. This lack of diversity among socio-demographic data may not represent the community of active adults, compromising our ability to generalize findings from our study to the population. The questionnaire was based online and so respondents may have searched for answers to the questions before selecting their answer.

## Conclusion

Only a small portion of the population are currently using dietary NO_3_^−^ supplements. The knowledge of current recommendations is generally poor among the population. An emphasis needs to be placed on knowledge translation because supplement education programs have limited effectiveness. Once the barriers to knowledge translation are identified and improved upon, the information will reach credible sources and flow down to the public accurately and effectively, resulting in the best possible health/performance outcomes from using dietary NO_3_^−^ supplements.

## Data availability statement

The raw data supporting the conclusions of this article will be made available by the authors, without undue reservation.

## Ethics statement

The studies involving humans were approved by Human Research Ethics Committee of The University of Queensland. The studies were conducted in accordance with the local legislation and institutional requirements. Written informed consent for participation in this study was provided by the participants’ legal guardians/next of kin.

## Author contributions

NM: Conceptualization, Data curation, Formal analysis, Investigation, Methodology, Writing – original draft, Writing – review & editing. PB: Conceptualization, Data curation, Formal analysis, Investigation, Methodology, Writing – original draft, Writing – review & editing. TP: Supervision, Writing – review & editing. ML: Supervision, Writing – review & editing.
