# Supplementary material for: Assessment of dietary nitrate supplementation: prevalence of use, knowledge, attitudes and beliefs among active Australians
Source: Front Nutr. 2023 Nov 2;10:1291431. doi: 10.3389/fnut.2023.1291431 (PMC10652790; doi:10.3389/fnut.2023.1291431)

## Supplementary Material for ‘*Assessment of dietary nitrate supplementation: prevalence of use, knowledge, attitudes and beliefs among active Australians’ by* McMahon NF, Brooker PG, Pavey T & Leveritt MD.

Correspondence to Paige G Brooker: [p.brooker@uq.edu.au](mailto:p.brooker@uq.edu.au)

## Online Survey


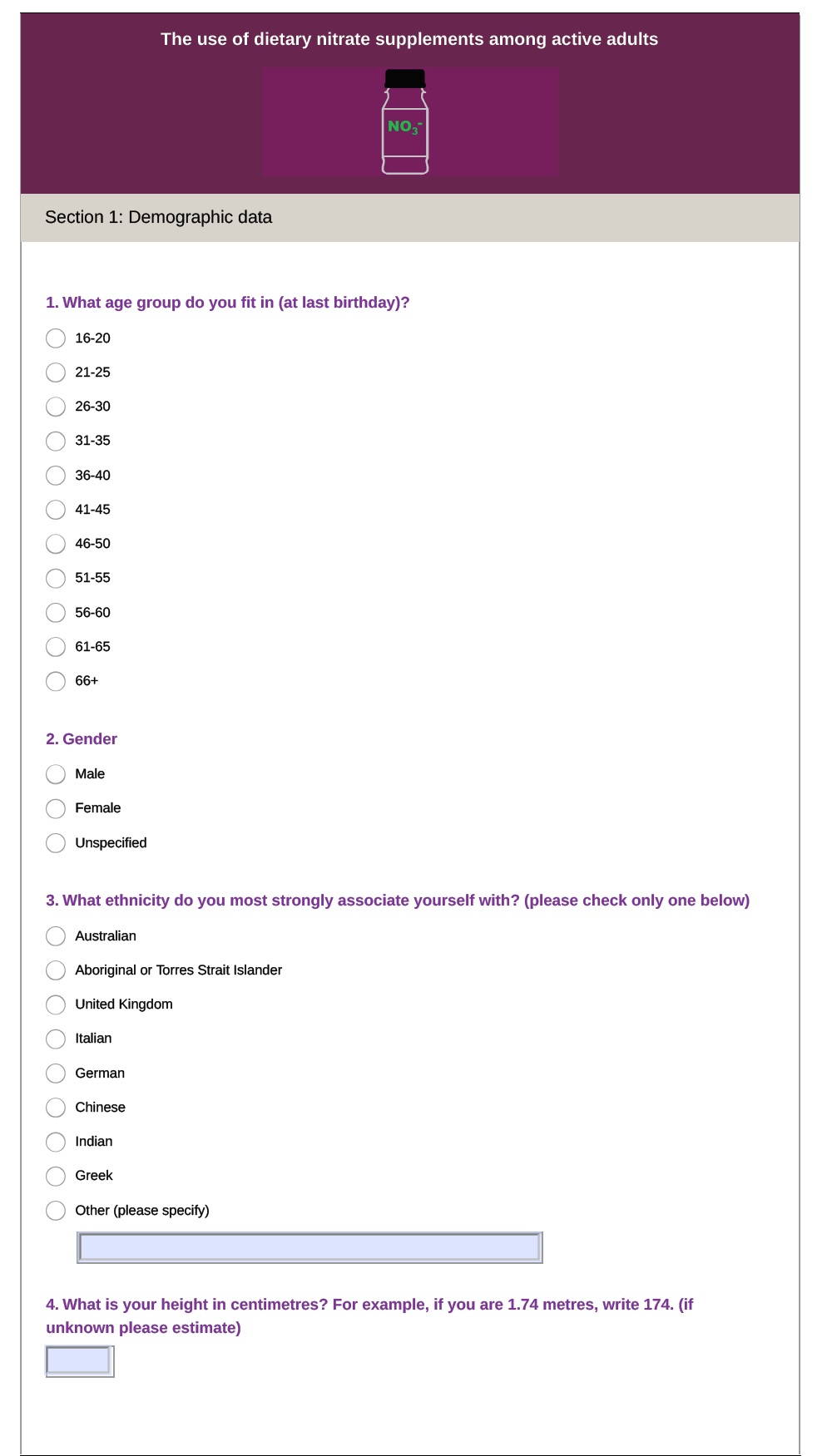


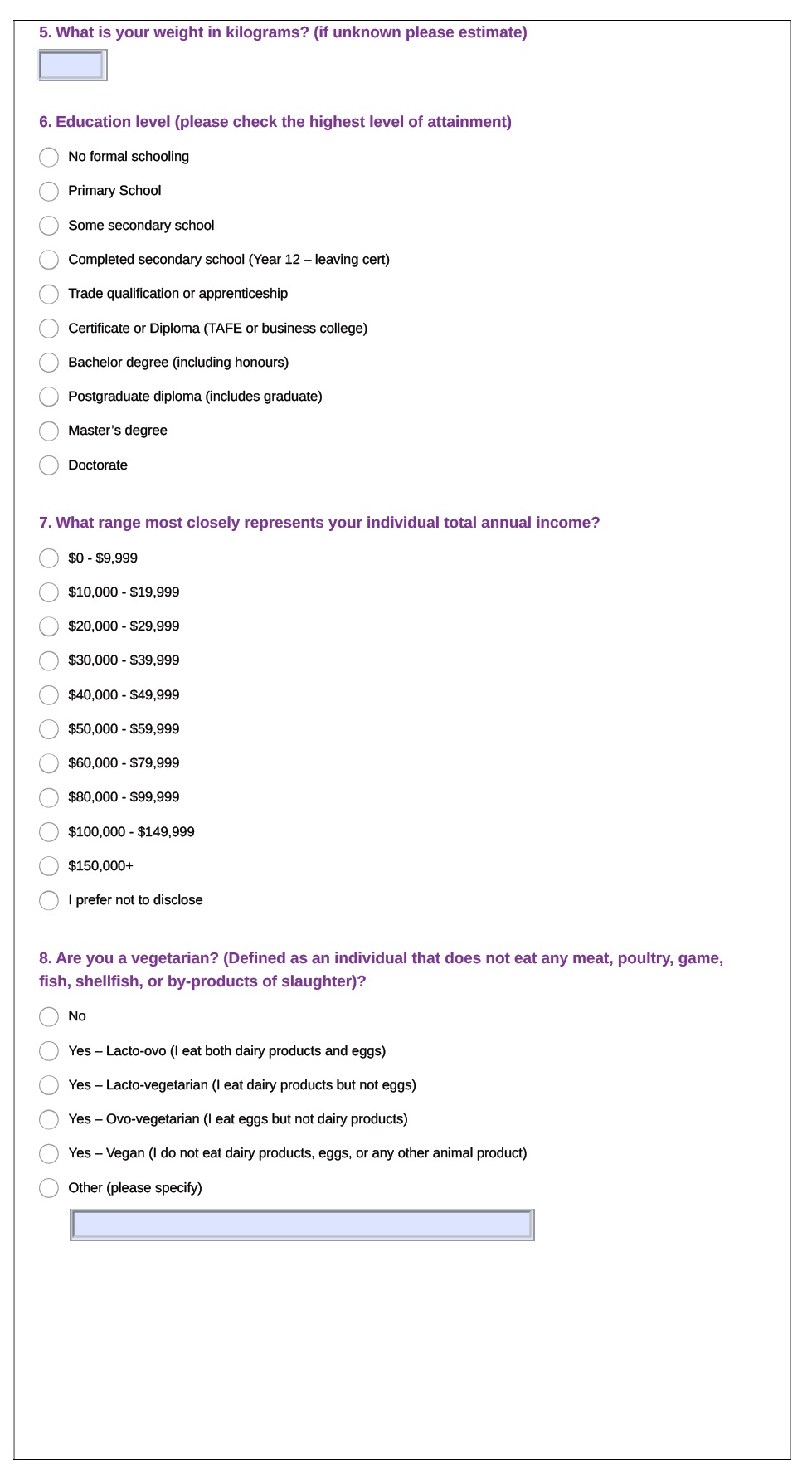


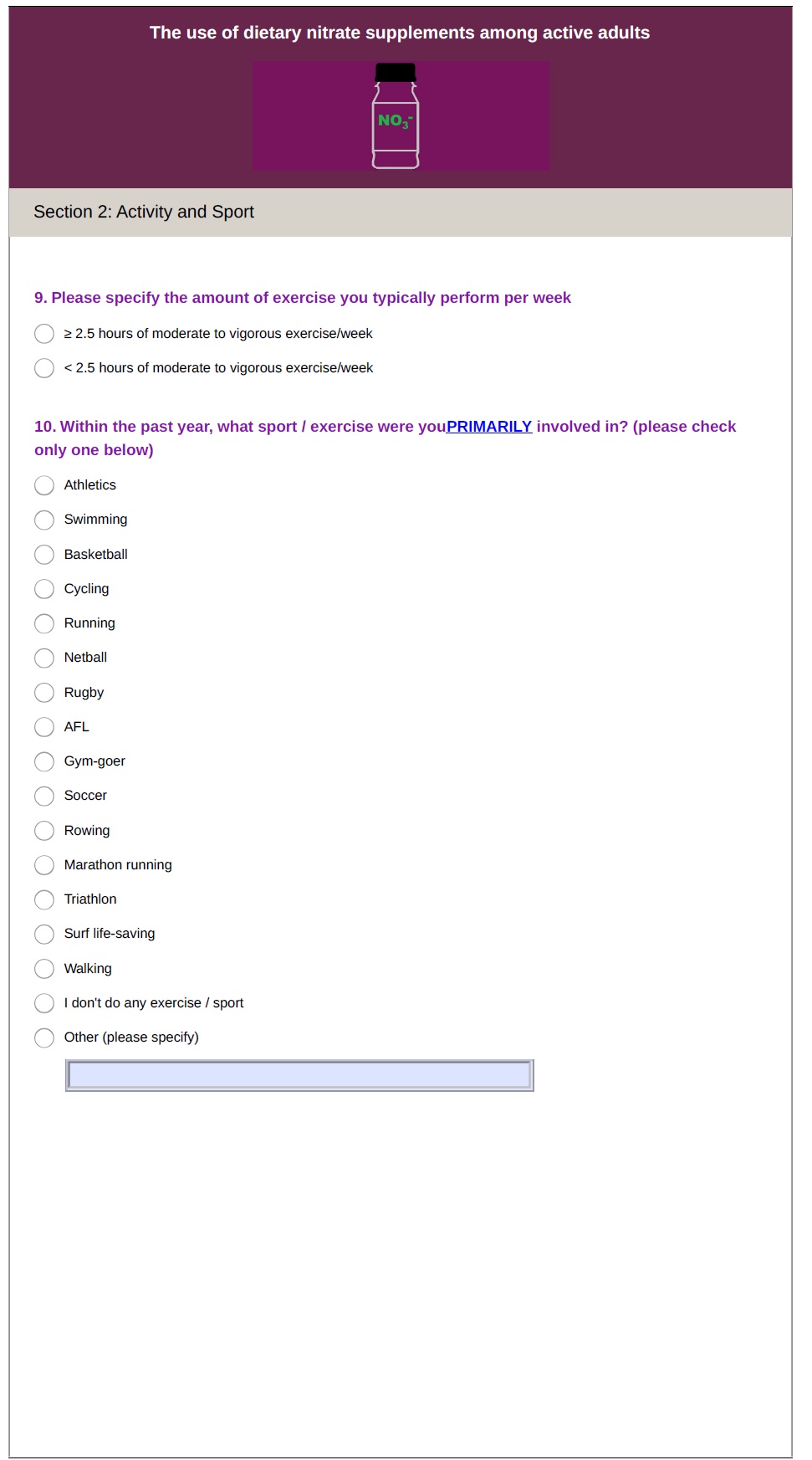


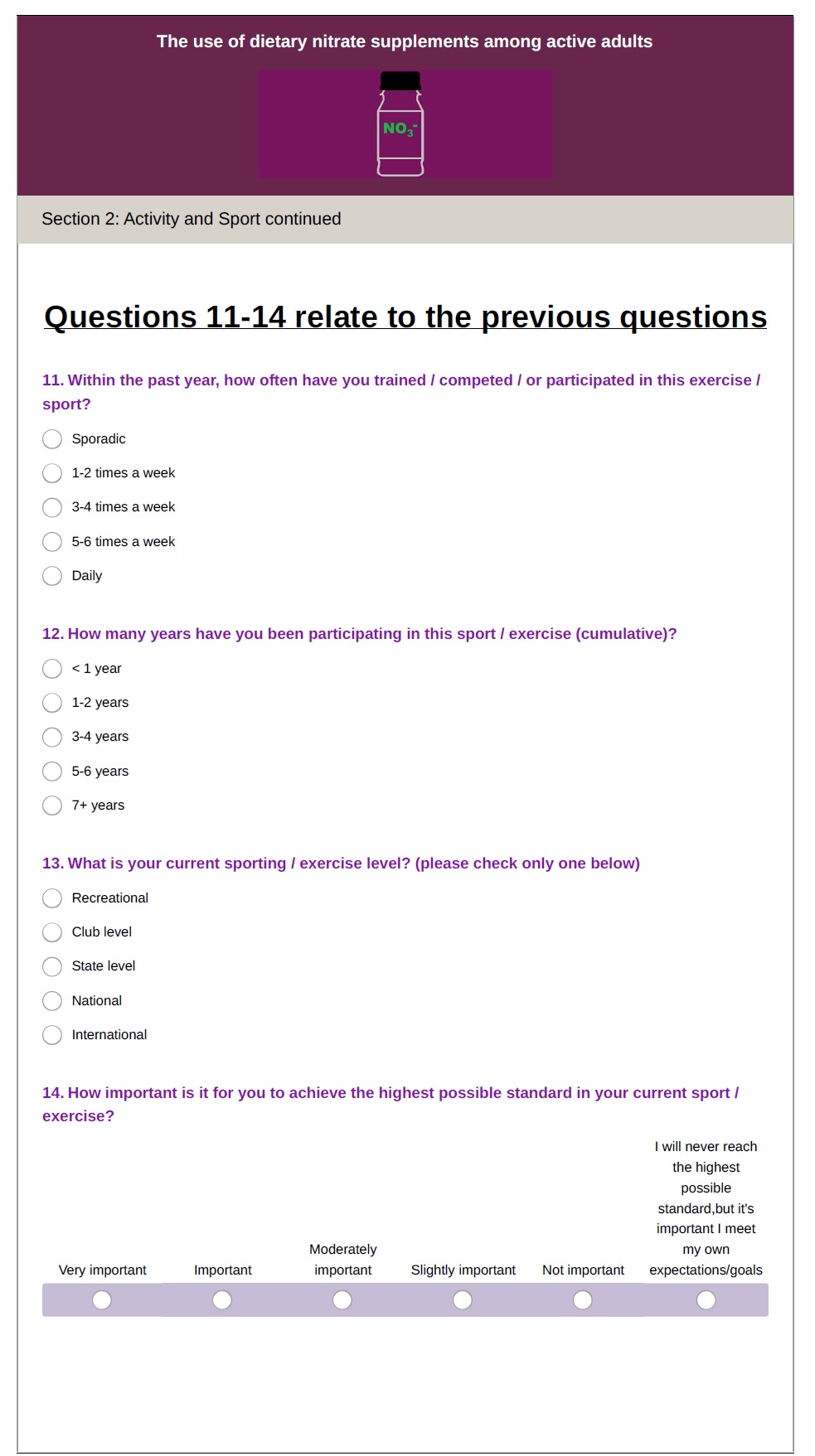


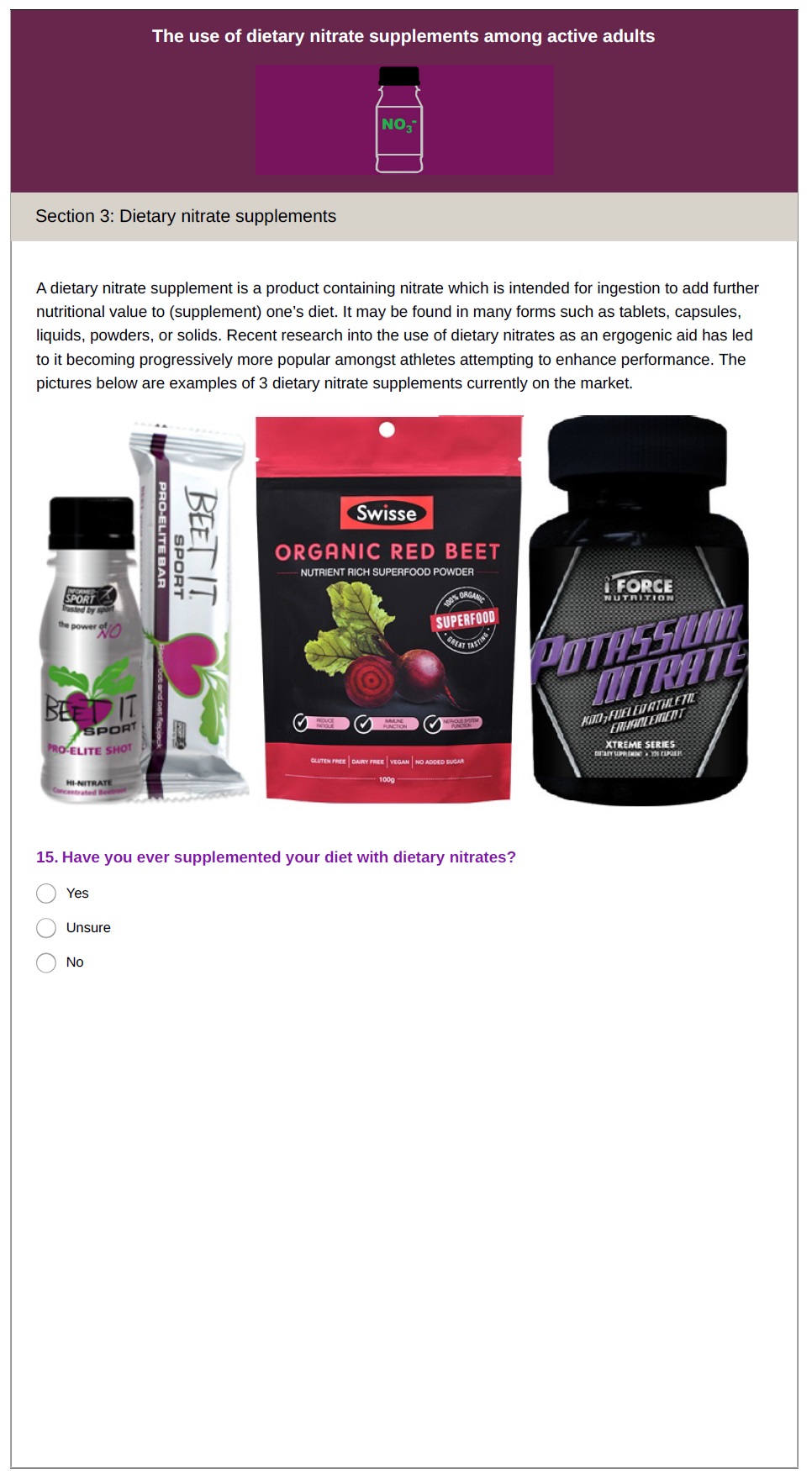


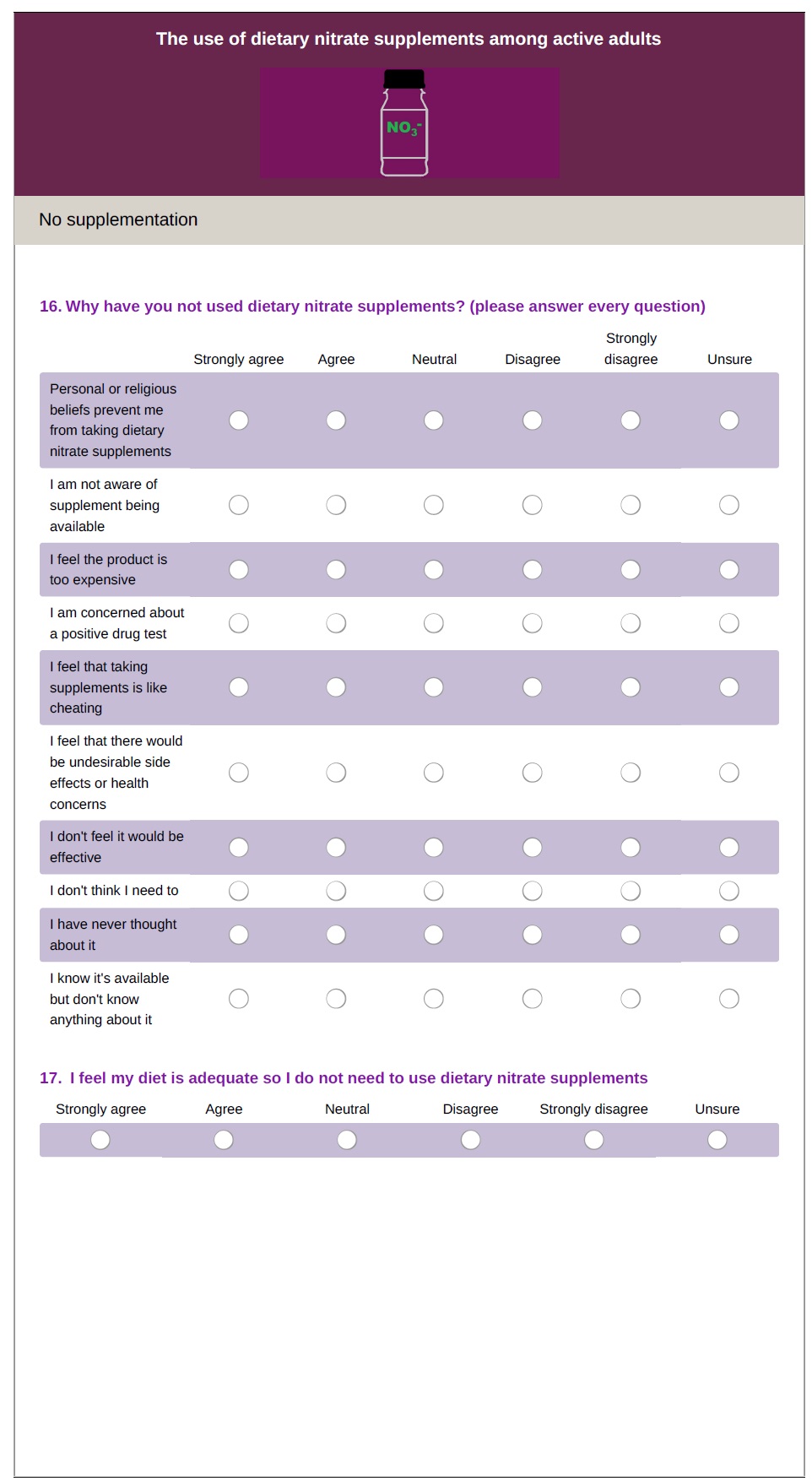


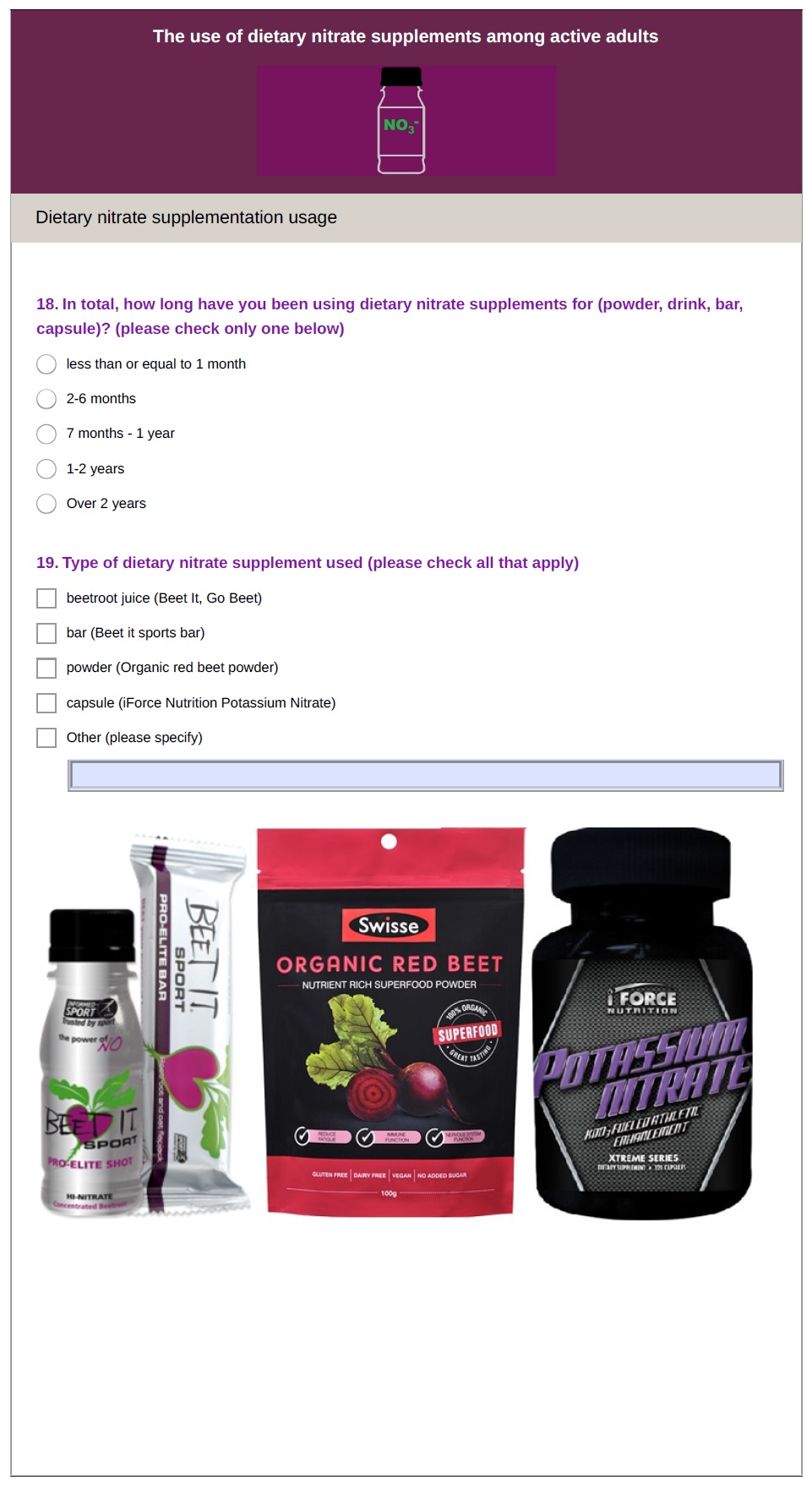


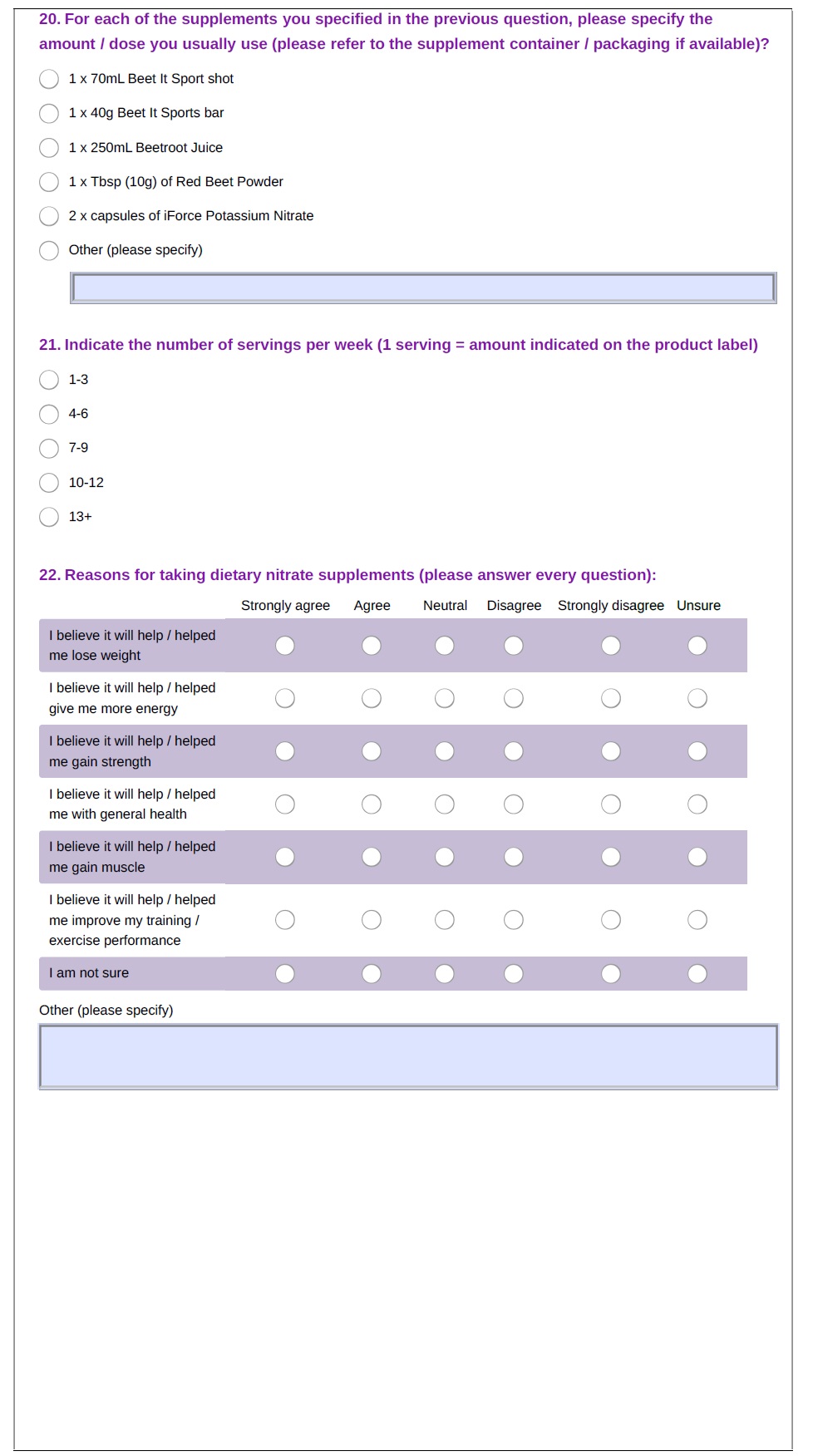


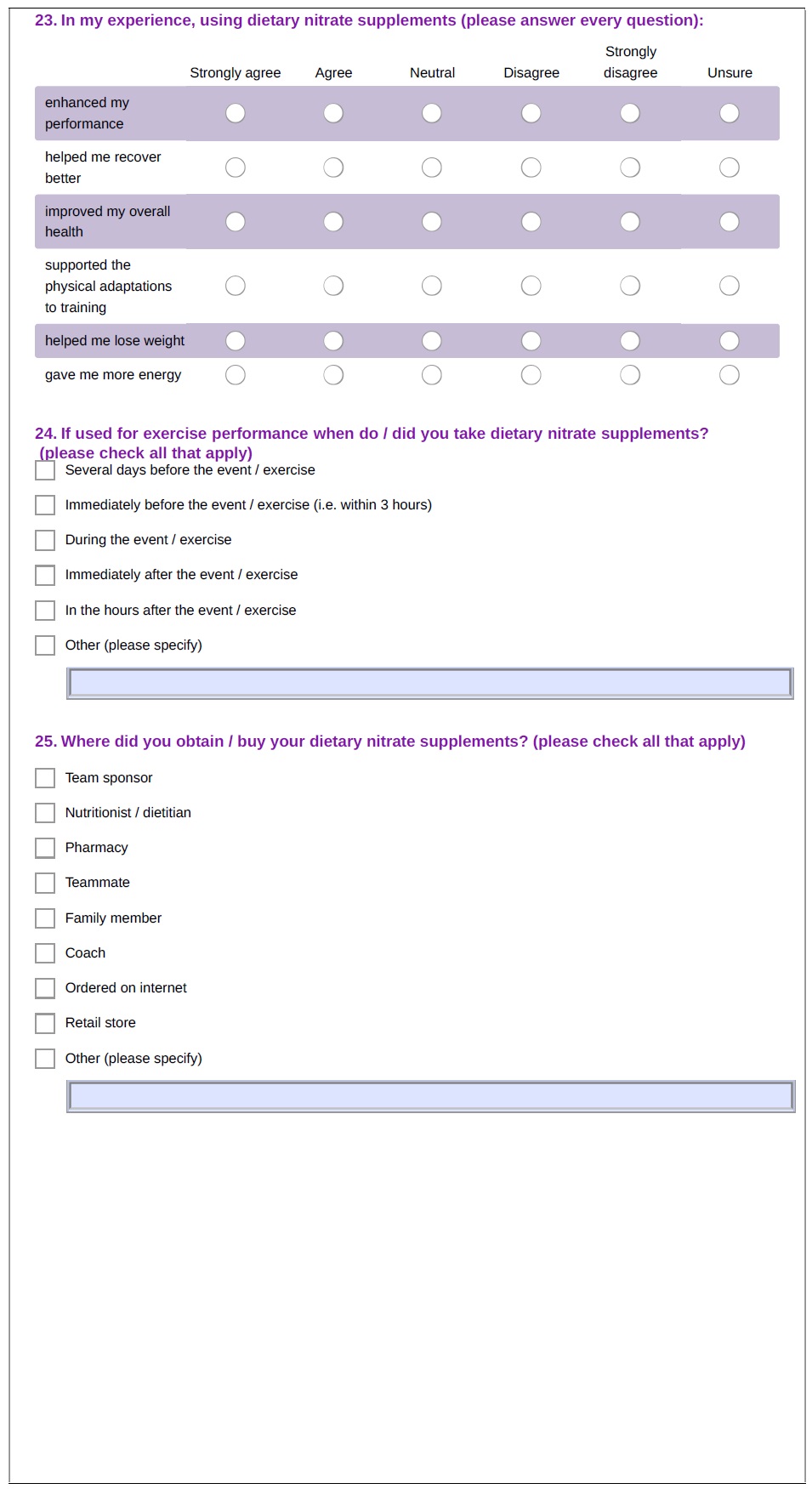


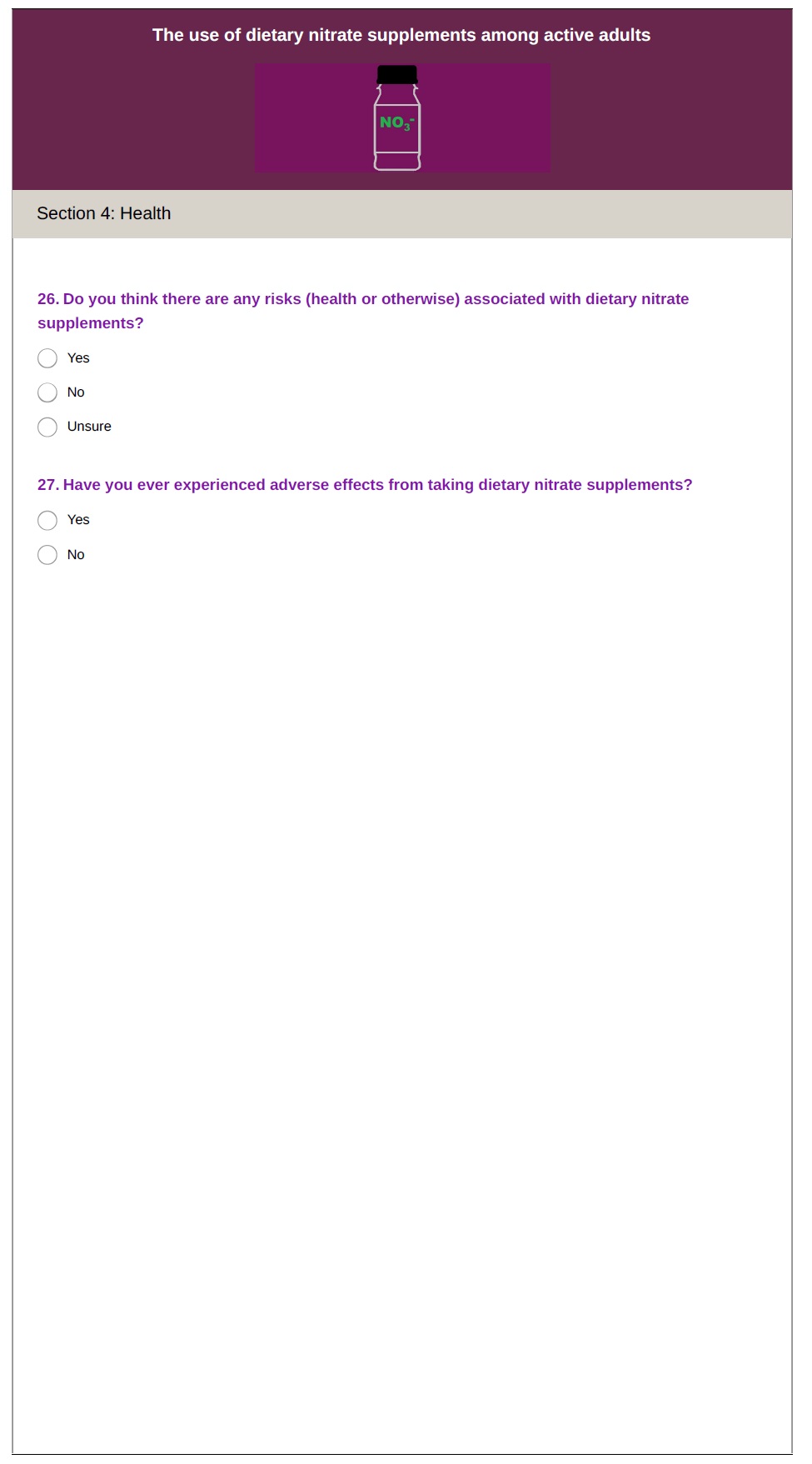


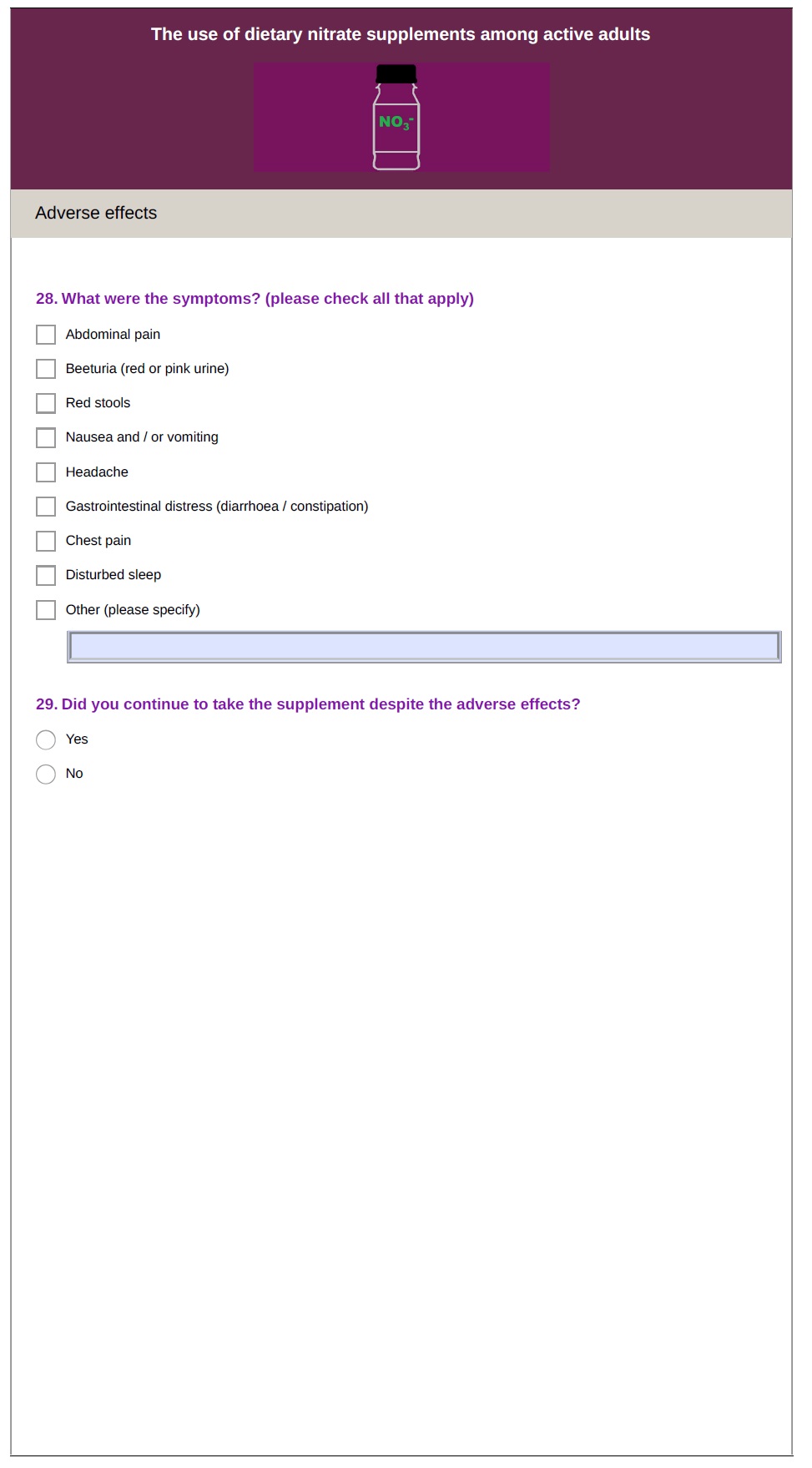


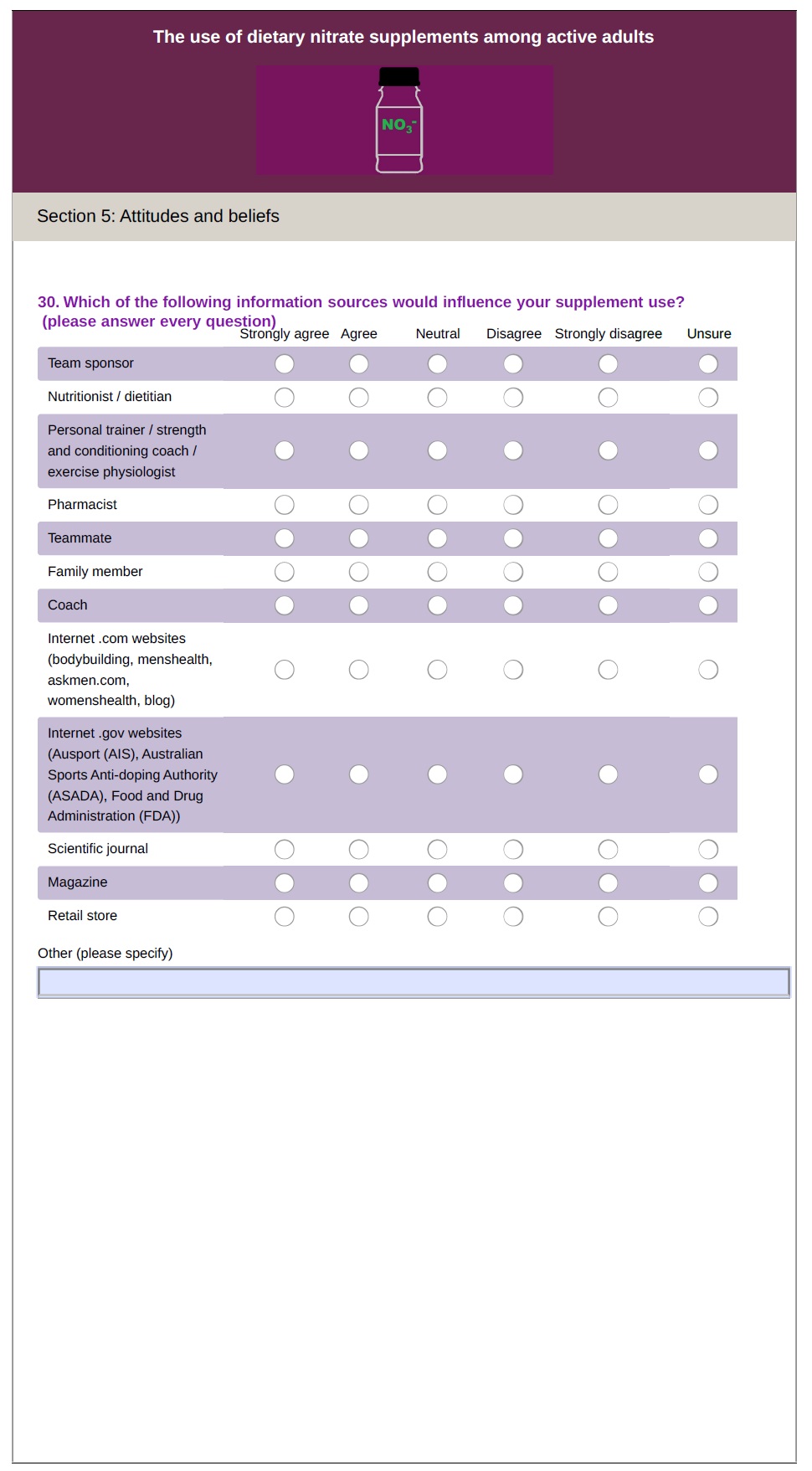


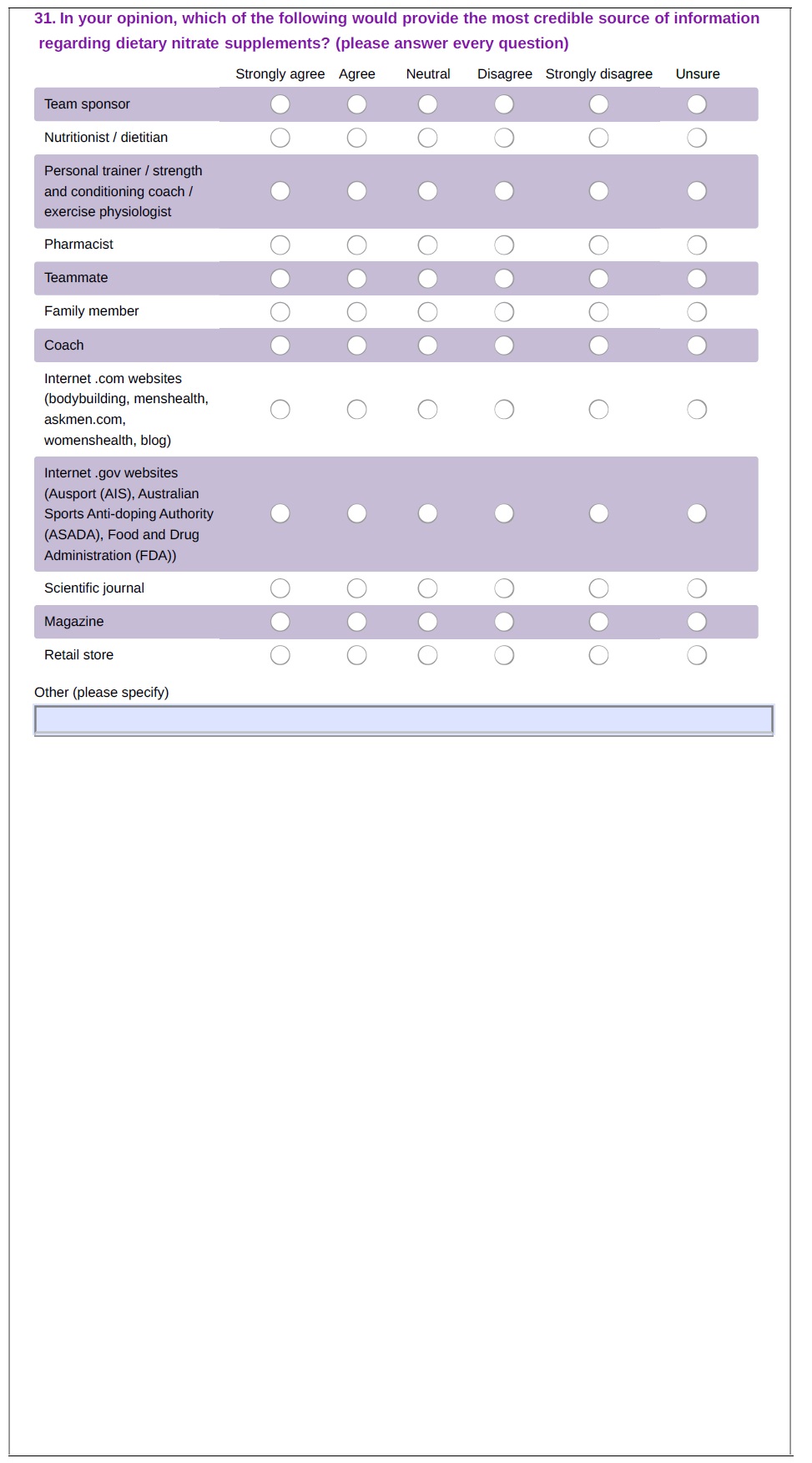


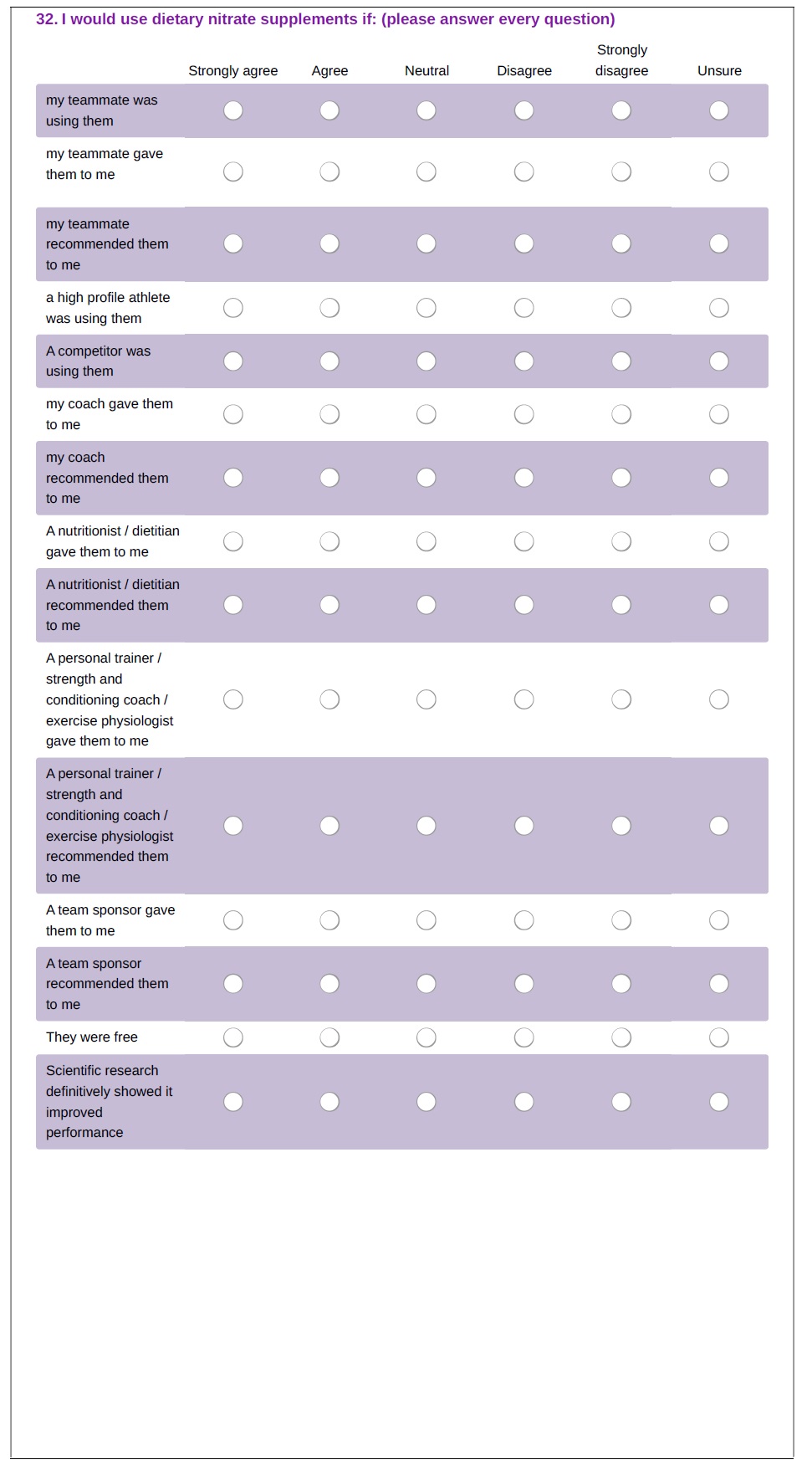


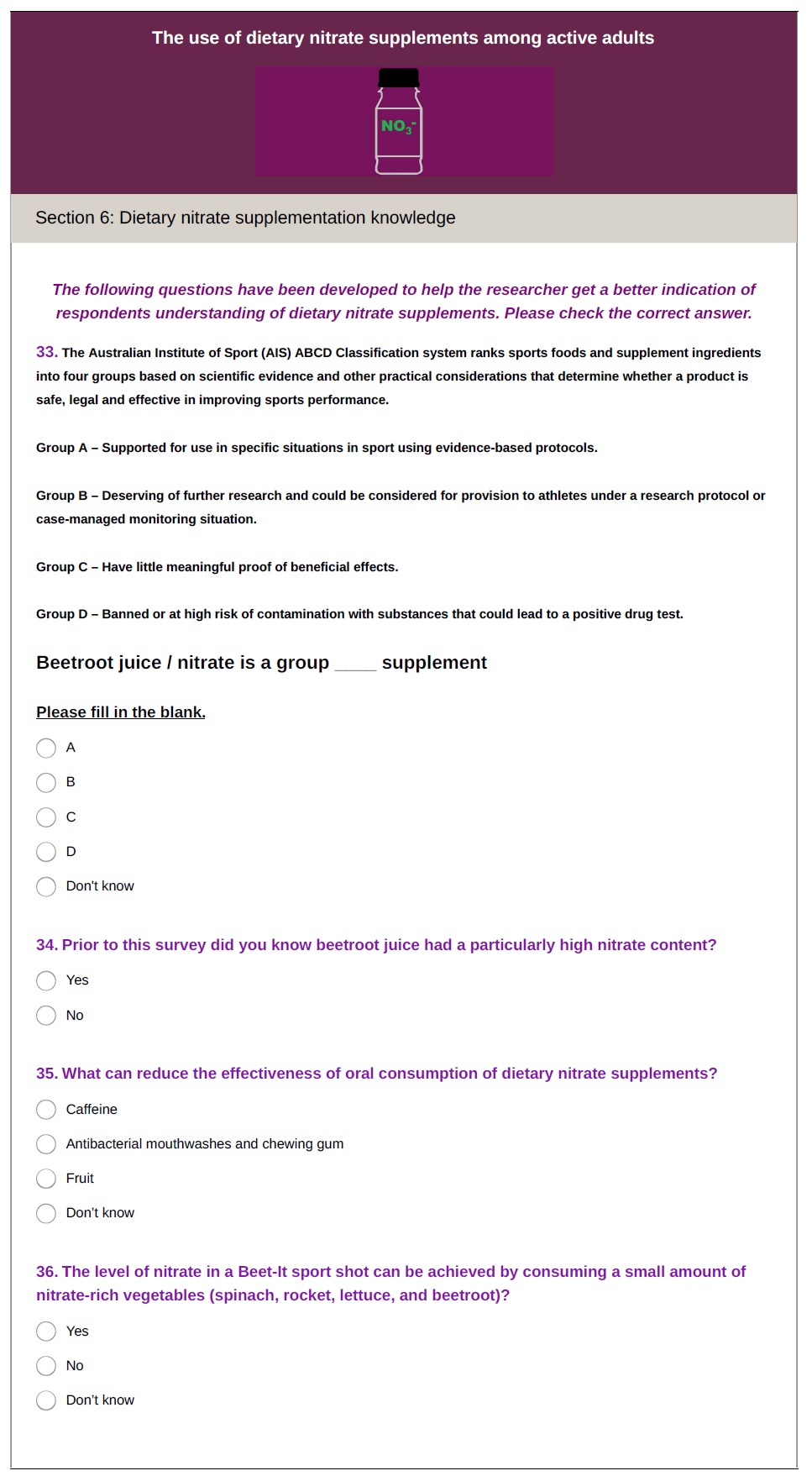


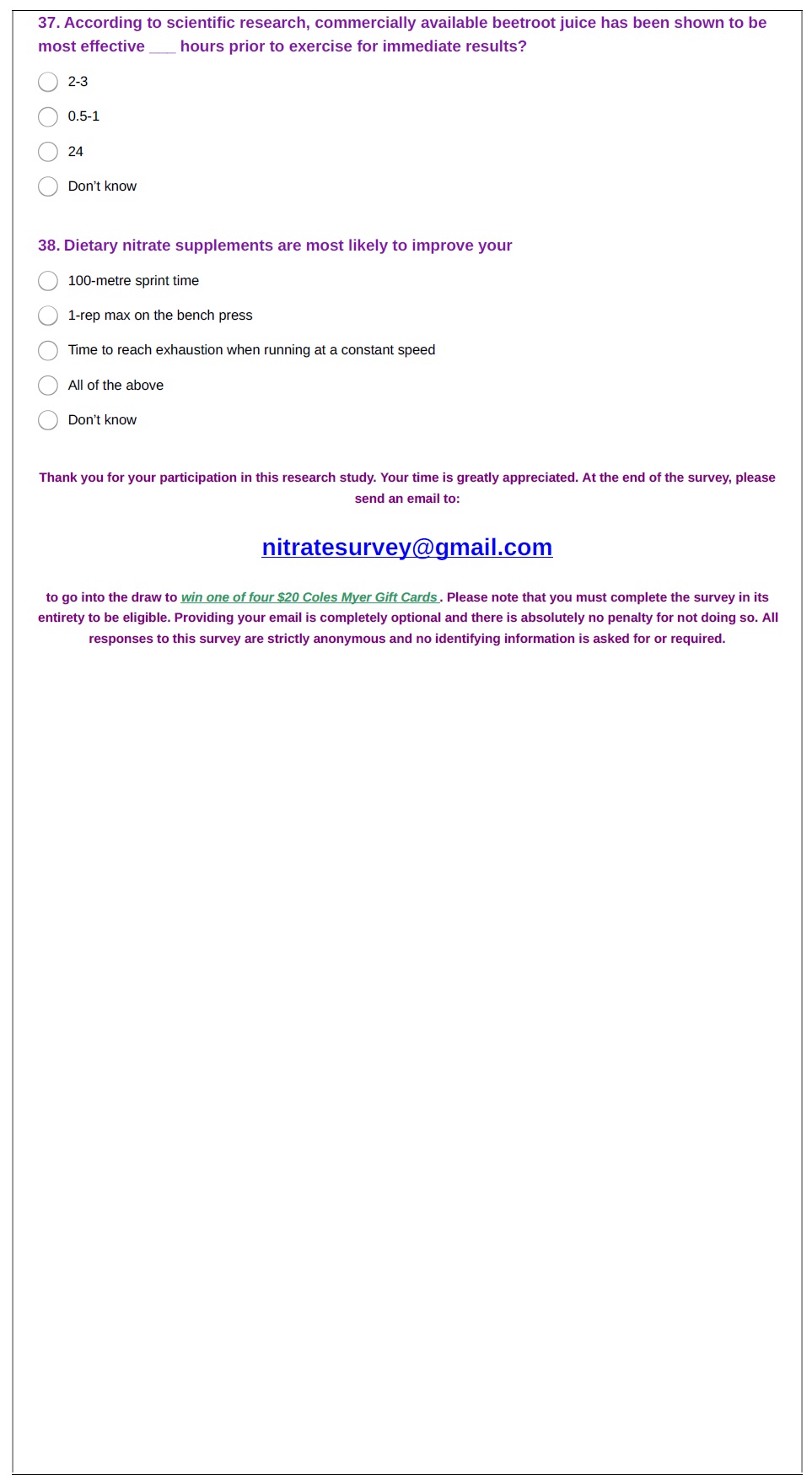

Supplement: Supplementary file 1 [file Data_Sheet_1.docx]
